# Glycemic variability evaluated by HbA1c rather than fasting plasma glucose is associated with adverse cardiovascular events

**DOI:** 10.3389/fendo.2024.1323571

**Published:** 2024-02-14

**Authors:** Lijuan Sheng, Guifang Yang, Xiangping Chai, Yang Zhou, Xin Sun, Zhenhua Xing

**Affiliations:** ^1^ Clinical Nursing Teaching and Research Section, Second Xiangya Hospital, Central South University, Changsha, China; ^2^ Department of Emergency Medicine, Second Xiangya Hospital, Central South University, Changsha, China; ^3^ Trauma Center, Second Xiangya Hospital, Central South University, Changsha, China; ^4^ Emergency Medicine and Difficult Diseases Institute, Second Xiangya Hospital, Central South University, Changsha, China; ^5^ College of nursing, Changsha Medical University, Changsha, China

**Keywords:** glycemic variability, HbA1c variability, fasting plasma glucose variability, type 2 diabetes mellitus, MACEs

## Abstract

**Background:**

Although studies have shown that glycemic variability is positively associated with an increased risk of cardiovascular disease, few studies have compared hemoglobin A1c (HbA1c) and fasting plasma glucose (FPG) variability with adverse cardiovascular events in patients with type 2 diabetes mellitus (T2DM).

**Methods:**

This was a *post hoc* analysis of the Action to Control Cardiovascular Risk in Diabetes (ACCORD) study. Cox proportional hazards models were used to explore the relationship between HbA1c or FPG variability and the incidence of major adverse cardiovascular events (MACEs).

**Results:**

In total, 9,547 patients with T2DM were enrolled in this study. During the median 4.6 ± 1.5 years follow-up period, 907 patients developed MACEs. The risk of MACEs increased in the HbA1c variability group in each higher quartile of HbA1c variability (P < 0.01). Compared with those in the first quartile of HbA1c variability, patients in the fourth quartile had a hazard ratio of 1.37 (Model 2, 95% confidence interval: 1.13–1.67) for MACEs. Higher FPG variability was not associated with a higher risk of MACEs in patients with T2DM (P for trend=0.28). A U-shaped relationship was observed between HbA1c and FPG variability, and MACEs. Glucose control therapy modified the relationship between HbA1c and MACEs; participants with higher HbA1c variability receiving intensive glucose control were more likely to develop MACEs (P for interaction <0.01).

**Conclusion:**

In adults with T2DM, the relationship between glycemic variability evaluated using HbA1c and FPG was U-shaped, and an increase in HbA1c variability rather than FPG variability was significantly associated with MACEs. The relationship between HbA1c variability and MACEs was affected by the glucose control strategy, and a higher HbA1c variability was more strongly associated with MACEs in patients receiving an intensive glucose control strategy.

## Introduction

Type 2 diabetes mellitus (T2DM) is a well-known independent risk factor for cardiovascular disease (CVD), and epidemiological studies have consistently demonstrated an association between the extent of hyperglycemia and the risk of these diseases (1, 2). However, several large randomized controlled clinical trials that targeted blood glucose or glycated hemoglobin A1c (HbA1c) to near-normal levels (intensive blood glucose-lowering therapy) did not reduce or even increase the risk of CVD compared to standard therapy among patients with diabetes mellitus (3–6). Thus, traditional glucose control based on HbA1c or fasting plasma glucose (FPG) levels may not be sufficient to predict long-term cardiovascular complications.

Recently, abnormal glycemic variability (GV) has gradually attracted the attention of researchers. Recent studies have shown that a greater GV is an independent risk factor for cardiovascular complications (7, 8). Both clinical GV and experimental findings suggest that the greater the glycemic variability, the higher the risk of cardiovascular complications (9, 10). However, data on the association between long-term variability in glycemic control and the risk of adverse cardiovascular outcomes are mixed (11). Some observational studies have indicated that glycemic variability is associated not only with macrovascular complications, such as CVD severity, but also with microvascular diabetes complications (12). Conversely, some previous studies failed to find a significant association between GV and major adverse cardiovascular events (MACEs). For example, Siegelaar SE et.al (13) found that in the HEART2D study, a decrease in glucose variability did not reduce cardiovascular event rates in patients with T2DM after acute myocardial infarction.

The magnitude of GV evaluated by HbA1c or FPG variability in relation to the risk of adverse cardiovascular events in T2DM patients is limited. Therefore, this study aimed to evaluate the prognostic value of several measures (HbA1c/FPG) of GV for the occurrence of separate cardiovascular complications in the ACCORD study.

## Methods

### Study population and data collection

The present investigation constituted a *post hoc* analysis of the ACCORD study, an encompassing randomized controlled trial involving 10,251 patients diagnosed with T2DM and afflicted with, or displaying a substantial propensity for, CVD. The primary objective of this study was to ascertain the potential enhancement of cardiovascular outcomes in patients with T2DM through intensified management of glycemic, hypertensive, and lipid profiles. Notably, the blueprint and principal findings of the trial have already been disseminated (14–16). The average age of participants diagnosed with T2DM was approximately 62 years, with a decade-long history of T2DM. Following a mean observation period of 3.7 years, the intervention was prematurely terminated due to the heightened peril of cardiac mortality associated with intensive blood glucose regulation. Consequently, all participants were transitioned to standard blood glucose management, and their progress was diligently monitored. Notably, intensified control of blood pressure and lipid levels failed to yield any improvement in CVD outcomes throughout the median follow-up duration of five years.

### Measures of glycemic variability

The assessment of GV involved the examination of fluctuations in HbA1c or FPG between visits for each participant. This assessment was conducted using repeated measures of HbA1c or FPG levels, spanning 8 months to 3 years during the follow-up period. The evaluation of variability was anchored at the 8-month mark, considering that the study intervention directly influenced fluctuations in glycemic markers in the initial months after participant enrollment. The determination of GV relied on core laboratory measurements of HbA1c and FPG levels. The accepted metric for assessing GV was denoted as the average successive variability (ASV), which was defined as the average absolute difference between consecutive values (17).

### Study outcomes

The principal measure of interest in this study was MACEs, which were delineated as composite outcomes comprising nonfatal myocardial infarction, nonfatal stroke, and/or cardiovascular mortality (18). The secondary endpoints of the study encompassed the individual components that constitute MACEs, namely cardiac death, nonfatal myocardial infarction (MI), and nonfatal stroke. The participants were subjected to regular follow-ups at intervals of 2–4 months. During the 4-month intervals, participants were queried regarding any pertinent medical events they may have experienced. MACEs were classified according to the Working Group of the Morbidity and Mortality Subcommittee.

### Variables

Participants underwent a series of activities in accordance with a standardized protocol, including the completion of questionnaires, physical examinations, and laboratory measurements. The covariates assessed at baseline included age, sex, race, glycemic control strategy (intensive or standard), CVD history, history of heart failure, educational status, depression status, smoking status, proteinuria, body mass index (BMI), duration of diabetes, alcohol consumption, cholesterol, triglycerides, low-density lipoprotein (LDL), high-density lipoprotein (HDL), HbA1c, FPG, systolic blood pressure (SBP), diastolic blood pressure (DBP), heart rate (HR), and glomerular filtration rate (GFR). Educational level was categorized as follows: lower than high school, high school graduate, college years, and college graduate or higher. Smoking status was classified into two categories: “never/former smoker” and “current smoker” (within the last 30 days). Alcohol consumption was categorized based on the weekly alcohol consumption.

### Statistical analysis

Categorical variables were compared using chi-square analysis, whereas continuous variables were compared using either analysis of variance or Mann–Whitney U tests, depending on the distribution type. Cox proportional hazards analyses were conducted to investigate the relationship between HbA1c and FPG variability, both as categorical and continuous variables, and adverse cardiovascular events. In Model 1, we adjusted for FPG, HbA1c, age, sex, race, and glucose control strategies. In Model 2, we adjusted for the covariates in Model 1 and the remaining variables listed in **Table 1**. In our analysis, we employ restricted cubic splines with four knots placed at the 25th, 50th, and 75th percentiles. This approach allowed us to flexibly model the association between HbA1c or FPG variability and adverse cardiovascular events using a Cox proportional hazards model. The models were adjusted for Model 2, considering relevant covariates. Subgroup and interaction analyses were conducted based on age, sex, race, duration of diabetes, and glucose control (intensive or standard). All statistical analyses were two-sided, and statistical significance was set at P < 0.05. The software used to perform the analyses was Stata/MP, version 17.0, developed by StataCorp.

**Table 1:** Basline characteristics of the patients.

MACEs	Yes	No	P-value
**N**	907	8640	
**Age, mean±SD;yr**	64.40 ± 7.12	62.57 ± 6.52	<0.001
**Female**	638 (70.34%)	5273 (61.03%)	<0.001
**Race**			0.001
No-White	291 (32.08%)	3250 (37.62%)	
White	616 (67.92%)	5390 (62.38%)	
**Glycemic control strategy**			0.043
Standard	484 (53.36%)	4306 (49.84%)	
Intensive	423 (46.64%)	4334 (50.16%)	
**History of CVD**	497 (54.80%)	2801 (32.42%)	<0.001
**History of heart failure**	89 (9.81%)	347 (4.02%)	<0.001
**Education**			0.002
Less than high school	158 (17.44%)	1208 (13.99%)	
High school graduate	230 (25.39%)	2287 (26.48%)	
Some college	318 (35.10%)	2838 (32.86%)	
College graduate or more	200 (22.08%)	2303 (26.67%)	
**Depression**	235 (25.91%)	1990 (23.04%)	0.052
**Current smoker**	134 (14.77%)	1166 (13.50%)	0.285
**Proteinuria**	219 (24.15%)	1654 (19.15%)	<0.001
**BMI, mean±SD; kg/m^2^**	31.99 ± 5.44	32.27 ± 5.39	0.130
**Duration of diabetes, mean±SD; yr**	12.15 ± 8.25	10.61 ± 7.46	<0.001
**Alcohol/week, mean ± SD;times**	1.00 ± 2.80	0.97 ± 2.69	0.752
**Cholesterol, mean ± SD; mg/dL**	185.65 ± 42.94	182.96 ± 41.65	0.066
**Triglyceride, mean ± SD; mg/dL**	199.44 ± 139.81	190.10 ± 150.91	0.075
**LDL, mean ± SD; mg/dL**	107.41 ± 34.88	104.46 ± 33.66	0.013
**HDL, mean ± SD; mg/dL**	39.97 ± 11.44	41.95 ± 11.43	<0.001
**HbA1c, mean ± SD; %**	8.48 ± 1.10	8.28 ± 1.04	<0.001
**FPG, mean ± SD; mg/dL**	144.00 ± 40.85	136.11 ± 35.10	<0.001
**SBP, mean ± SD; mmHg**	137.73 ± 18.19	136.07 ± 16.88	0.005
**DBP, mean ± SD; mmHg**	73.26 ± 11.49	75.04 ± 10.49	<0.001
**HR, HR, mean ± SD; bpm**	72.08 ± 12.32	72.65 ± 11.64	0.168
**GFR, mean ± SD; ml/min/1.73 m^2^)**	86.95 ± 27.18	91.51 ± 27.17	<0.001

## Results

### Baseline characteristics of population

**Figure 1:**
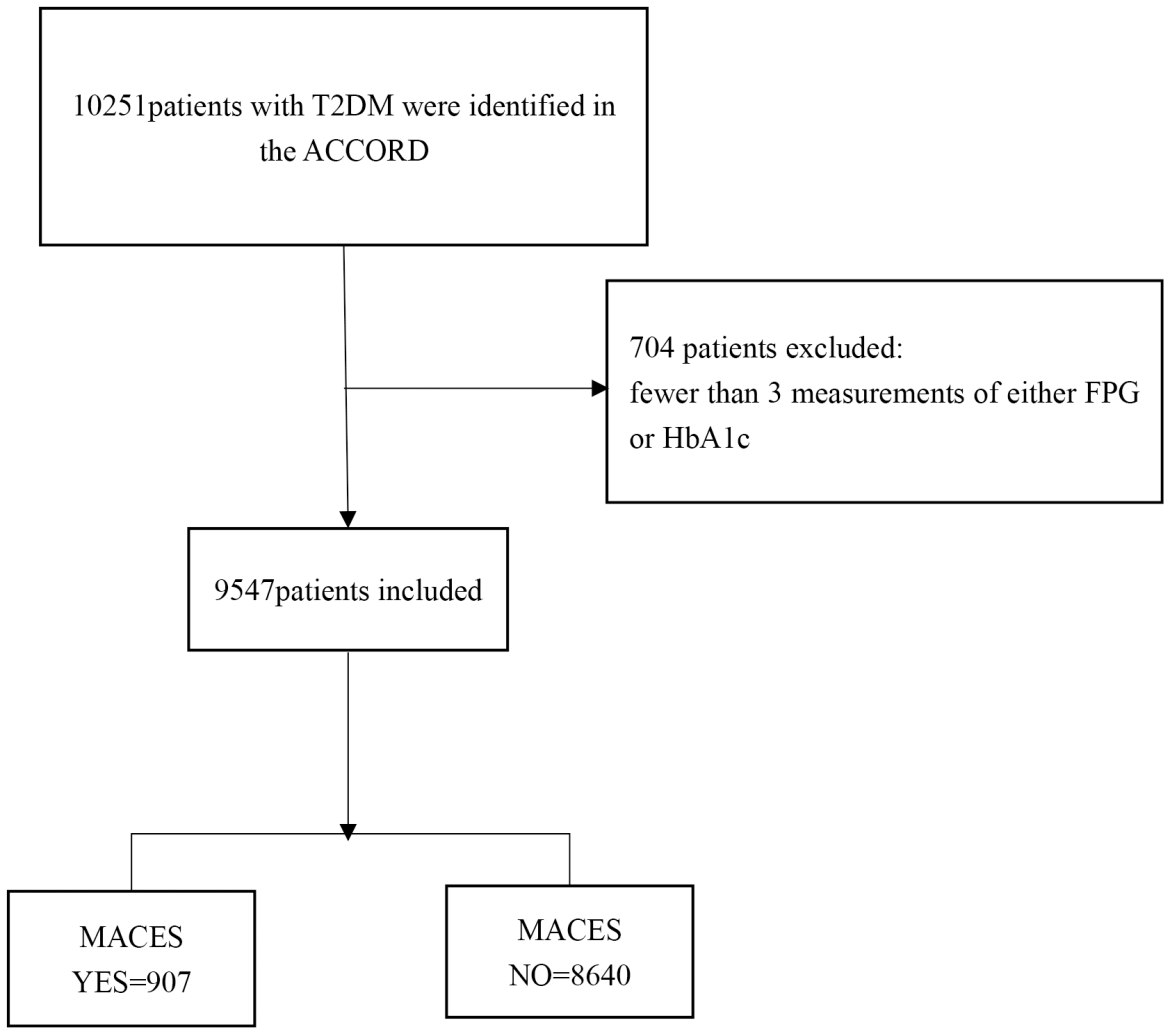
Flow chart.

Of the initial 10,251 participants enrolled in the ACCORD, 9,547 were included in the analysis (**Figure 1**). A subset of 704 participants was excluded from the analysis due to having fewer than 3 measurements of either FPG or HbA1c. During the median 4.6 ± 1.5 years follow-up period, 907 participants (9.5%) developed MACEs (331 cardiac death [3.5%], 631 non-fatal MI [6.6%], and 197 non-fatal strokes [2.1%]). The baseline characteristics of the selected participants according to MACEs status are shown in **Table 1**. The MACEs group showed significant differences in age, white race, standard glycemic control, history of CVD, history of heart failure, education, proteinuria, duration of diabetes, LDL, HbA1C, FPG, and SBP. No statistically significant differences were detected in depression, current smoking status, BMI, alcohol/week, cholesterol, triglyceride, or HR.

### The relationship between glycemic variability and MACEs

The association between GV evaluated by HbA1c and FPG levels and the risk of MACEs is presented in **Table 2**. Each 1 standard deviation (SD) increase in HbA1c variability was associated with a 11% higher risk of MACEs (Model 2, 95% CI 1.02–1.19). However, FPG variability was not associated with MACEs (Model 2, HR 1.07,95% CI 0.98–1.16).The risk of MACEs increased in the HbA1c variability group with each higher quartile of HbA1c variability in the first model. Compared with those in the first quartile of HbA1c variability, patients in the fourth quartile had an HR of 1.37 (Model 2, 95% CI 1.13–1.67, P for trend < 0.01) for MACEs. Participants in the fourth quartile of FPG variability had an HR of 1.19 (95% CI 0.97–1.45, P for trend = 0.28, Model 2) for MACEs. A higher FPG variability was not associated with a higher risk of MACEs in patients with T2DM.

**Table 2:** Relationship between glycemic variability and MACEs in different models.

	Incidence rate $	HR (95%CI)	
		Model 1	Model 2
MACEs			
HbA1c variability			
1	16.7	Ref	Ref
2	20.9	1.25(1.03-1.50)	1.20(0.99-1.45)
3	22.5	1.29(1.07-1.56)	1.17(0.97-1.42)
4	28.2	1.46(1.20-1.77)	1.37(1.13-1.67)
**P for trend**		<0.01*	<0.01*
**Per SD increase**		1.13(1.03-1.22)	1.11(1.02-1.19)
FPG variability			
1	17.0	Ref	Ref
2	20.7	1.25(1.03-1.50)	1.17(0.97-1.41)
3	20.4	1.29(1.07-1.56)	1.01(0.83-1.22)
4	30.1	1.46(1.20-3.04)	1.19(0.97-1.45)
**P for trend**		<0.01*	0.28
**Per SD increase**		1.13(1.05-1.21)	1.07(0.98-1.16)

$ per 1,000 person-years. * P value<0.05

Model 1: fasting plasma glucose, HbA1c, age, sex, race, glucose control strategy.

Model 2: covariates in model 1 and other remaining variables listed in **Table 1**.

**Figure 2:**
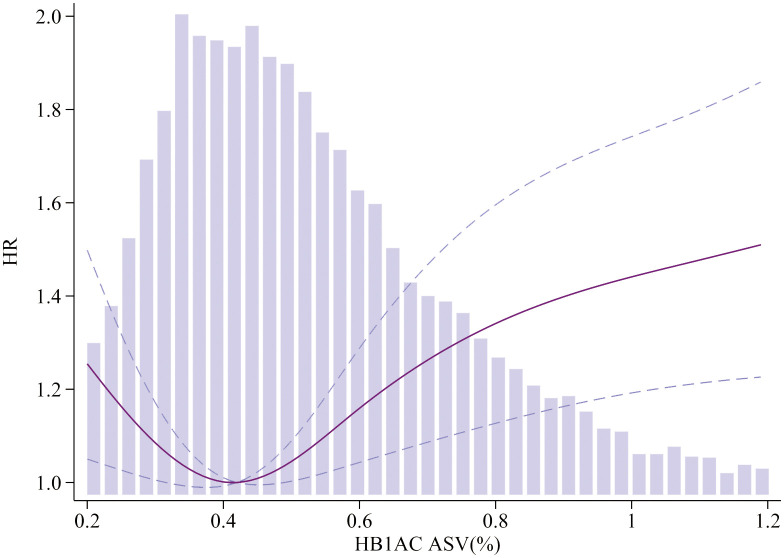
Association of HbA1c ASV and MACEs. Smooth spline curves of HbA1c for the estimation of risk of MACEs after adjusting multivariate rates. MACEs major adverse cardiovascular events.

**Figure 3:**
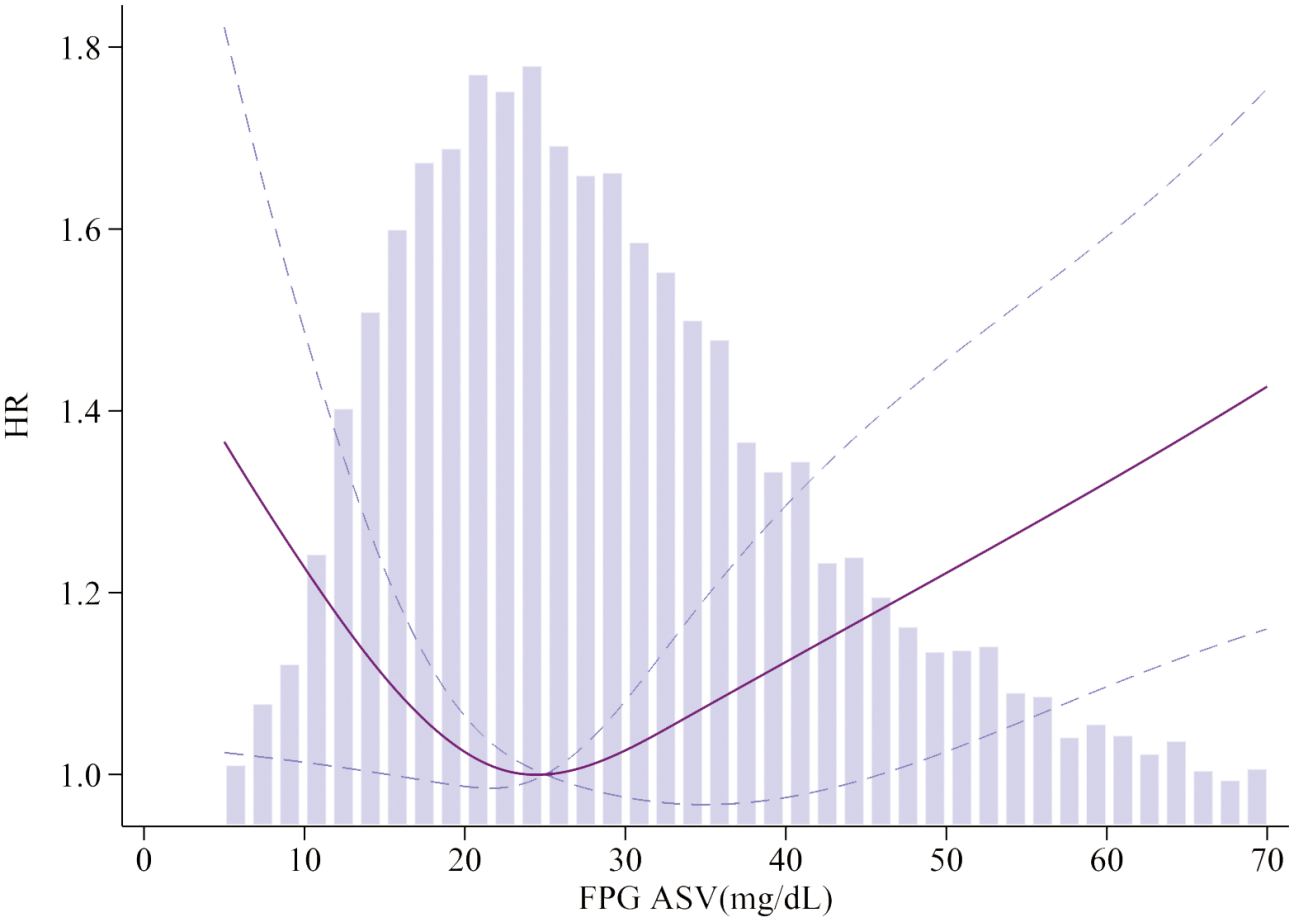
Association of FPG ASV and MACEs. Smooth spline curves of HbA1c for the estimation of risk of MACEs after adjusting multivariate rates. MACEs major adverse cardiovascular events.

To visualize the nonlinear association between HbA1c/FPG variability and the incidence of MACEs, restricted cubic splines were used for flexible modeling (**Figures 2**, **3**). A U-shaped relationship existed between HbA1c/FPG variability and MACEs.

### Second endpoints

Higher GV was positively associated with nonfatal MI in both the HbA1c and FPG variability groups. Patients in the higher quartiles of HbA1c variability had a higher risk of nonfatal stroke. However, the same relationship was not found for cardiac death in either the HbA1c/FPG variability group or nonfatal stroke in the FPG variability group (**Table 3**).

**Table 3:** Relationship between glycemic variability and second endpoints in different models.

	Incidence rate $	HR (95%CI)	
		Model 1	Model 2
HbA1c variability			
Cardiac Death			
1	6.7	Ref	Ref
2	5.2	0.77(0.56-1.07)	0.75(0.54-1.04)
3	6.6	0.93(0.68-1.27)	0.83(0.61-1.14)
4	8.4	0.97(0.70-1.34)	0.91(0.66-1.27)
P for trend		0.92	0.71
Per SD increase		0.92(0.77-1.09)	0.88(0.72-1.05)
Non-fatal MI			
1	8.2	Ref	Ref
2	14.4	1.73(1.35-2.22)	1.66(1.30-2.14)
3	13.7	1.61(1.25-2.08)	1.48(1.14-1.91)
4	16.5	1.82(1.40-2.37)	1.76(1.34-2.30)
P for trend		<0.01	<0.01
Per SD increase		1.18(1.09-1.28)	1.16(1.07-1.27)
Non-fatal Stroke			
1	2.4	Ref	Ref
2	3.9	1.11(0.67-1.83)	1.09(1.00-1.60)
3	3.9	1.54(0.96-2.46)	1.41(0.88-2.67)
4	5.6	1.99(1.24-3.22)	1.86(1.14-3.03)
P for trend		<0.01	<0.01
Per SD increase		1.18(1.00-1.39)	1.15(0.97-1.37)
FPG variability			
Cardiac Death			
1	6.3	Ref	Ref
2	6.6	1.00(0.73-1.37)	1.00(0.73-1.38)
3	5.3	0.71(0.51-0.98)	0.66(0.47-0.92)
4	8.6	0.83(0.60-1.15)	0.81(0.58-1.12)
P for trend		0.09	0.06
Per SD increase		1.07(0.92-1.25)	1.03(0.88-1.20)
Non-fatal MI			
1	8.9	Ref	Ref
2	11.5	1.25(0.97-1.62)	1.24(0.95-1.60)
3	14.5	1.51(1.18-1.94)	1.42(1.10-1.82)
4	17.9	1.65(1.27-2.14)	1.48(1.13-1.93)
P for trend		<0.01	<0.01
Per SD increase		1.17(1.08-1.27)	1.11(1.02-1.21)
Non-fatal Stroke			
1	3.1	Ref	Ref
2	3.4	1.07(0.69-1.68)	1.03(0.66-1.61)
3	2.4	0.69(0.43-1.13)	0.63(0.39-1.03)
4	5.6	1.39(0.88-2.19)	1.11(0.70-1.77)
P for trend		0.75	0.94
Per SD increase		1.10(0.92-1.32)	1.03(0.86-1.25)

$ per 1,000 person-years. * P value<0.05

Model 1: fasting plasma glucose, HbA1c, age, sex, race, plasma glucose control strategy.

Model 2: covariates in model 1 and other remaining variables listed in **Table 1**.

### Sensitivity and subgroup analysis

**Figure 4:**
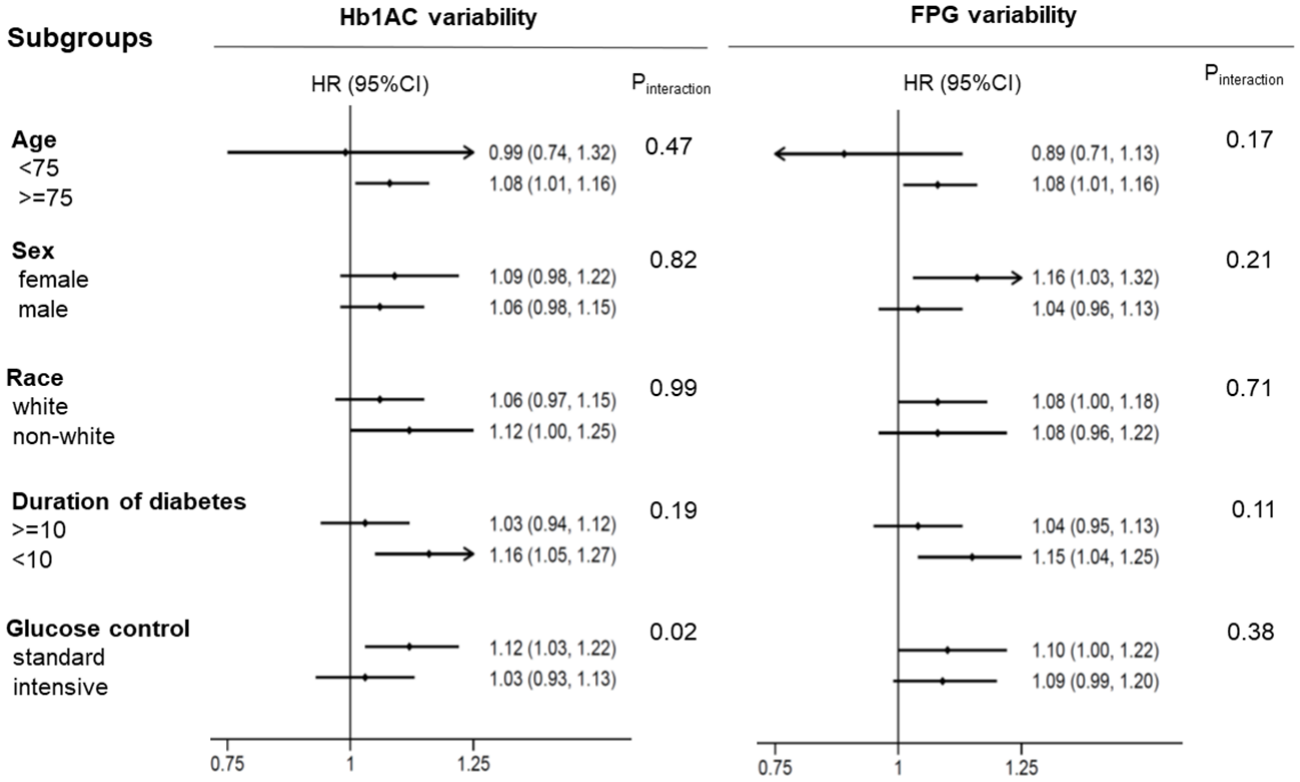
The HR per 1-SD ASV increase in HbA1c/FPG variability for MACEs. Each stratification was adjusted for all factors in Model 2, except for the stratification factor itself. MACEs major adverse cardiovascular events.

We further verified the association between glycemic variability, as evaluated by HbA1c and FPG levels, and the risk of MACEs. The association between HbA1c and FPG variability and the incidence of MACEs in the different subgroups is shown in **Figure 4**. The results showed that glucose control strategy played an interactive role in the association between HbA1c variability and MACEs incidence. Higher HbA1c variability in participants receiving intensive glucose control was more likely to lead to MACEs.

## Discussion

In our *post hoc* analysis that focused on patients with an average 10-year history of T2DM and a heightened risk of adverse cardiovascular events, we observed a noteworthy disparity in the association between GV and cardiovascular outcomes. Notably, GV was assessed using HbA1c levels as opposed to FPG, demonstrated a significant correlation with adverse cardiovascular events.

GV is gradually gaining recognition as an important parameter in the evaluation of glycemic control. A growing body of research has revealed a strong link between GV and diabetes-related complications, particularly adverse cardiovascular events (19). Consistent with our findings, there is increasing recognition of HbA1c variability as an unfavorable prognostic factor in T2DM. The ADVANCE study further supports this notion by demonstrating that a higher HbA1c variability is associated with an elevated risk of vascular events and mortality (20). Notably, this study employed a randomized controlled trial design that allowed the monitoring of treatment adherence, providing robust evidence in support of the observed association. However, it is important to know that the HbA1c variability does not evaluate short term glucose variations which may have an even greater impact in MACE. Short-term glucose variations, as captured by Continuous Glucose Monitoring (CGM) and Mean Amplitude of Glycemic Excursions (MAGE), can provide valuable insights into glycemic patterns that may impact cardiovascular outcomes.

Currently, a standardized definition of HbA1c variability has not been established. Various studies have utilized different measures to express variability, including standard deviation (SD), coefficient of variation (CV), and average successive variability (ASV), which are calculated based on all HbA1c measurements. The choice of measurement may vary depending on the study and its objectives (21). Indeed, considering the progressive nature of T2DM and the natural tendency of HbA1c levels to rise over time, relying solely on the SD or CV may result in inflated values relative to the mean. This can occur without adequately capturing the true fluctuations in HbA1c levels (22). Therefore, alternative measures, such as ASV, may provide a more accurate representation of HbA1c fluctuations in such scenarios. In addition, Mone P et al. (23) also found that stress hyperglycemia ratio on hospital admission significantly and independently increases the risk of rehospitalization for chest pain in ischemia with nonobstructive coronary arteries patients.

The observed lack of an association between FPG variability and CVD events appears to contradict the findings of previous studies. A recent cohort study conducted in the general population, comprising 53,607 participants with a mean age of 49.1 years and a 5-year follow-up period, demonstrated different outcomes. This study revealed that even after adjusting for the mean FPG value and other relevant covariates, individuals in the highest quartile of FPG variability exhibited increased risks of CVD (26% higher) and mortality (46% higher) than those in the lowest quartile (24). However, our findings do not align with the results of the aforementioned study and several factors may have contributed to this disparity. First, the population included in the ACCORD study specifically consisted of individuals with T2DM, whereas the study by Jang et al. encompassed the general population. Additionally, variations in the methodologies employed to estimate variability could also contribute to differences in the findings. It is crucial to consider these variations in the population and methodology when interpreting and comparing study results. In addition, Echouffo-Tcheugui JB et al. (25) in the ALLHAT Study, showed no excess risk of CVD in individuals with high FBG variability in the United States, which further supports our conclusion.

The precise mechanisms by which increased GV leads to an elevated risk of adverse outcomes are not yet fully understood; however, several hypotheses have been proposed. One possible explanation relates to the pathophysiological alterations associated with glycemic fluctuations compared to stable glucose levels. These fluctuations can contribute to higher levels of inflammatory cytokines, which, in turn, may lead to endothelial dysfunction. These effects have been observed not only in individuals with diabetes but also in those with normal blood glucose levels (26). Additionally, both *in vivo* and *in vitro* experimental studies have revealed that glucose fluctuations, compared with stable glucose levels, are associated with significantly elevated levels of oxidative stress markers. Increased oxidative stress serves as a major catalyst for adverse cardiovascular events, further emphasizing the potential link between GV and negative cardiovascular outcomes (27). Furthermore, a clinical study reported that high GV is linked to an increased risk of thrombosis. This finding suggests a direct role of GV in promoting the development of adverse cardiovascular events through thrombotic mechanisms (28).

To the best of our knowledge, the present study represents the first observation of a U-shaped relationship among HbA1c levels, FPG variability, and MACEs in patients with T2DM. Additionally, we assessed and compared the predictive value of HbA1c and FPG variability for adverse cardiovascular events in our study cohort. Our findings suggest that glycemic variability, evaluated based on HbA1c levels, is associated with an increased risk of adverse cardiovascular events. Furthermore, we conducted this study using a relatively large sample size, which enhanced the robustness of the outcomes compared to previous research. In addition, we performed subgroup analyses based on various population characteristics and study features. This comprehensive approach significantly enhances the reliability and accuracy of our conclusions. Despite providing valuable insights, this study had certain limitations that should be acknowledged. First, owing to the focus on the T2DM population, the generalizability of the results may be limited. Therefore, a large-scale, adequately powered, prospective, multicenter study is required to validate this hypothesis. An important limitation of this study was the absence of information on dietary factors that could potentially influence clinical outcomes. It would be intriguing to investigate the impact of diet on the development of future cardiovascular adverse events, as diet could serve as a significant confounding factor. Addressing these limitations in future research will enhance our understanding and applicability of these findings in a broader context. Finally, ACCORD was completed in 2008 when most patients were treated with metformin, sulfonylurea, and insulin whereas the T2DM treatment mode has changed significantly with the use of gliptins, glutides, and gliflozins. Therefore, applicability of the results to modern practice is questionable.

## Conclusion

In adults diagnosed with T2DM, our study revealed a U-shaped relationship between GV, as assessed by HbA1c and FPG. Notably, an increase in HbA1c variability, rather than in FPG variability, was significantly associated with MACEs.

## Data availability statement

The original contributions presented in the study are included in the article/supplementary materials, further inquiries can be directed to the corresponding author/s.

## Ethics statement

Ethical approval was not required for the study involving humans in accordance with the local legislation and institutional requirements. Written informed consent to participate in this study was not required from the participants or the participants’ legal guardians/next of kin in accordance with the national legislation and the institutional requirements.

## Author contributions

GY: Writing – original draft. LS&XC: Supervision, Writing – review & editing. YZ: Software, Writing – review & editing. XS: Formal analysis, Writing – review & editing. ZX: Investigation, Writing – review & editing.
